# Expression and possible role of Smad3 in postnecrotizing enterocolitis stricture

**DOI:** 10.1136/wjps-2021-000289

**Published:** 2022-01-05

**Authors:** Rui Chen, Chengjie Lv, Xiaoxia Zhao, Dong Ma, Dengming Lai, Yun Zhao, Luyin Zhang, Jinfa Tou

**Affiliations:** 1 Department of Neonatal Surgery, Zhejiang University School of Medicine Children's Hospital, Hangzhou, People's Republic of China; 2 National Center for clinical medicine of children's health and disease, Hangzhou, People's Republic of China; 3 Department of Pathology, Zhejiang University School of Medicine Children's Hospital, Hangzhou, People's Republic of China; 4 Pediatric Surgery, Affiliated Hangzhou First People's Hospital, Zhejiang University School of Medicine, Hangzhou, People's Republic of China

**Keywords:** gastroenterology, neonatology, surgery, plastic, cell biology

## Abstract

**Objective:**

To investigate the expression of Smad3 (mothers against decapentaplegic homolog 3) protein in postnecrotizing enterocolitis stricture and its possible mechanism of action.

**Methods:**

We used immunohistochemistry to detect the expression characteristics of Smad3 and nuclear factor kappa B (NF-κB) proteins in human postnecrotizing enterocolitis stricture. We cultured IEC-6 (crypt epithelial cells of rat small intestine) in vitro and inhibited the expression of Smad3 using siRNA technique. Quantitative PCR, western blotting, and ELISA were used to detect the changes in transforming growth factor-β1 (TGF-β1), NF-κB, tumor necrosis factor-α (TNF-α), vascular endothelial growth factor (VEGF), and zonula occludens-1 (ZO-1) messenger RNA (mRNA) and protein expressions in IEC-6 cells. CCK8 kit and Transwell cellular migration were used to detect cell proliferation and migration. Changes in epithelial–mesenchymal transition (EMT) markers (E-cadherin and vimentin) in IEC-6 cells were detected by immunofluorescence technique.

**Results:**

The results showed that Smad3 protein and NF-κB protein were overexpressed in narrow intestinal tissues and that Smad3 protein expression was positively correlated with NF-κB protein expression. After inhibiting the expression of Smad3 in IEC-6 cells, the mRNA expressions of NF-κB, TGF-β1, ZO-1, and VEGF decreased, whereas the mRNA expression of TNF-α did not significantly change. TGF-β1, NF-κB, and TNF-α protein expressions in IEC-6 cells decreased, whereas ZO-1 and intracellular VEGF protein expressions increased. IEC-6 cell proliferation and migration capacity decreased. There was no significant change in protein expression levels of EMT markers E-cadherin and vimentin and also extracellular VEGF protein expression.

**Conclusions:**

We suspect that the high expression of Smad3 protein in postnecrotizing enterocolitis stricture may promote the occurrence and development of secondary intestinal stenosis. The mechanism may be related to the regulation of TGF-β1, NF-κB, TNF-α, ZO-1, and VEGF mRNA and protein expression. This may also be related to the ability of Smad3 to promote epithelial cell proliferation and migration.

**Key messages**

**What is already known about this subject?**

- The most common complication after conservative treatment in patients with necrotizing enterocolitis (NEC) is intestinal stenosis.
- The mechanism of intestinal stenosis may be related to intestinal tissue ischemia, inflammation, fibrous hyperplasia, and so on.
- Activation of TGF-β1/Smad3 (transforming growth factor-β1/mothers against decapentaplegic homolog 3) signaling pathway is an important mechanism in promoting intestinal fibrosis.
- Smad3 is the most direct effector protein in intestinal fibrosis.

**What are the new findings?**

- We found that intestinal fibrosis was the primary manifestation of intestinal stenosis secondary to NEC.
- Smad3 protein is overexpressed in intestinal stenosis secondary to NEC.
- There was a synergistic relationship between Smad3 protein expression and collagen fiber expression in intestinal stenosis secondary to NEC.
- Smad3 protein can regulate the expression of TGF-β1, nuclear factor kappa B, tumor necrosis factor-α, zonula occludens-1, and vascular endothelial growth factor in intestinal epithelial cells.
- Smad3 protein can promote intestinal epithelial cell proliferation and migration.

**How might it impact on clinical practice in the foreseeable future?**

- Exploring Smad3 inhibitors may help prevent intestinal fibrosis and may reduce the length of the narrow bowel.

## Introduction

Neonatal necrotizing enterocolitis (NEC) is one of the most common digestive tract infectious diseases in premature infants and is the main cause of death in premature infants. With the progress in premature infant medicine, the survival rate of premature infants has increased; however, the incidence of NEC has increased^1^ and the related complications have also increased. Intestinal stenosis is a common secondary NEC lesion.^2^ Currently, there are few basic studies on the mechanism of secondary intestinal stenosis in NEC. However, the specific mechanism of action is unclear. This may be related to the increase in collagen fiber expression and to the deposition of extracellular matrix (ECM) caused by inflammatory reactions in intestinal wall tissues.^3–5^

Smad3 protein (mothers against decapentaplegic homolog 3), a member of the Smad protein family, is a key link in intracellular transforming growth factor-β1 (TGF-β1) signal transduction, acting as a TGF-β1 receptor activator protein and being the most direct effector that leads to tissue fibrosis.^6^ Smad3 proteins have been shown to promote the progression of organ fibrosis in studies on inflammatory bowel disease (IBD), pulmonary fibrosis, liver fibrosis, and intestinal fibrosis caused by IBD.^7–9^ Currently, the treatment of NEC secondary to intestinal stenosis can only be done through surgical treatment because the resection of the narrow segment of the intestinal tube can relieve the obstruction.^2 5^ Through this treatment route, children inevitably lose more intestinal tissue. If intestinal stenosis is reduced, the chance of surgery or resection of intestinal tissue may be reduced, which would positively impact the prognosis of children.

Whether Smad3 protein plays a promoting role in the process of postnecrotizing enterocolitis stricture has not been studied. Based on this, the expression characteristics of the Smad3 protein in secondary intestinal stenosis and its possible mechanism of action on secondary intestinal stenosis were preliminarily studied in vivo and in vitro; moreover, these experimental data were intended to assist in understanding whether Smad3 can be used as a target for the prevention of NEC secondary to intestinal stenosis.

We obtained the pathological characteristics of postnecrotizing enterocolitis stricture by H&E and Masson staining, and we further obtained the expression characteristics of Smad3 protein by immunohistochemistry (IHC) to analyze the relationship between Smad3 protein and nuclear factor kappa B (NF-κB) inflammatory signaling pathway. After inhibiting the expression of Smad3 in IEC-6 (crypt epithelial cells of rat small intestine) in vitro, the changes in inflammatory and fibrosis molecular indexes NF-κB, TGF-β1, tumor necrosis factor-α (TNF-α), and vascular endothelial growth factor (VEGF) were detected to analyze whether Smad3 protein regulates these fibrosis indexes. Intestinal mucosal barrier function and epithelial–mesenchymal transition (EMT) are also two important mechanisms of intestinal inflammation, fibrosis, and stenosis.^10^ We examined the effects of Smad3 on the proliferation and migration of intestinal epithelial cells, on the expression of tight junction protein zonula occludens-1 (ZO-1), and on EMT to explore the mechanism of Smad3 affecting postnecrotizing enterocolitis stricture.

## Materials and methods

### Clinical specimens

Fifty-six paraffin specimens of children with intestinal stenosis treated with neonatal surgery in our hospital from January 2018 to August 2019 were collected. All 56 children had a definite history of NEC and were diagnosed with intestinal stenosis based on intraoperative and postoperative pathologies. The narrow-segment intestinal tissue was selected as the experimental group and the incised margin intestinal tissue was used as the control group.

### Chemicals and materials

Rabbit anti-human restructuring anti-Smad3 (Abcam USA, ab40854), rabbit anti-human NF-κB p65 antibody C-20 (Santa Cruz Biotechnology USA, sc-372), rabbit anti-human TGF-β1 antibody V (Santa Cruz Biotechnology USA, sc-146), mouse monoclonal antibody TNF-α (Abcam USA, ab1793), and rabbit anti-human ZO-1 antibody (Abcam USA, ab221547) were used. CCK8 (Cell Counting Kit-8) kit (Tongren Chemical, Japan), VEGF-ELISA kit (Elabscience), Smad3-siRNA (Invitrogen), and EMT detection markers E-cadherin and vimentin were purchased from Beijing Keystone Life Technology. All other reagents were used at an analytical grade.

### Histological examination

All specimens were fixed with 10% neutral buffered formalin, routinely dehydrated, and continuously sliced after paraffin embedding (approximately 4 μm thick slices) for H&E (hematoxylin-eosinstaining) and Masson staining.^11 12^ Staining was performed with H&E and Masson according to the manufacturer’s instructions. Masson staining of each section was taken under a 5×10 magnification microscope, and the images should include the mucosal layer, submucosal layer, and part of the muscular layer. The collagen fiber area was measured using ImageJ software.

### IHC analysis (EnVision two-step method)

IHC analysis was performed to determine the protein level of Smad3 and NF-κB p65. Sections of paraffin-embedded intestinal canal were dewaxed and washed with phosphate buffered solution (PBS), and then 3% H_2_O_2_ solution was added, which was followed by incubation in blocking buffer for 10 min. Intestinal canal sections were then incubated with rabbit anti-human restructuring anti-Smad3 (1:100) and polyclonal rabbit anti-human NF-κB p65 (1:100), incubated for 60 min at 37°C, and then incubated with a secondary antibody against rabbit IgG antibody-HRP (horseradish peroxidase) polymer, which was followed by washing with PBS. Finally, tissue slides were stained with diaminobenzidine and hematoxylin for counterstaining and were imaged using a microscope.^13^

IHC results were scored semiquantitatively: 0 points meant that positive cells accounted for less than 5%, 1 point meant that positive cells accounted for 5%–25%, 2 points meant that positive cells accounted for 26%–50%, and 3 points meant that positive cells accounted for ≥51%. According to the staining intensity score, 0 points means no staining of cells, 1 point means light yellow cells, 2 points means brown yellow cells, and 3 points means brown cells. The sum of the score of the percentage of stained cells and the score of staining intensity was used to determine the results of staining. A score of 0–1 was considered negative, and a score of ≥2 was considered positive.^14^ The Smad3 positive staining area was quantified using the average optical density (AOD) function in the ImageJ analyzer software.

### Cell culture and cell transfection

The cell line is IEC-6 (rat small intestine epithelium cell 6) which was purchased from the American type culture collection (ATCC) cell bank. IEC-6 cells were cultured according to the supplier’s recommendations. IEC-6 cells were cultured in Dulbecco's modified eagle medium (DMEM) supplemented with 10% fetal bovine serum, 1% penicillin/streptomycin, and 0.1 U/mL bovine insulin as described.^15^ Smad3 was knocked down by small interfering RNA (siRNA) in IEC-6 cells according to the manufacturer’s instructions. IEC-6 cells were seeded in six-well plates, and then a specific Smad3 siRNA and negative control (NC) siRNA were transfected with Lipofectamine RNAiMAX. After 48 hours, the total RNA of cells was extracted for quantitative PCR detection.

### Real-time quantitative PCR analysis

IEC-6 cells were inoculated into a six-well cell culture plate at a density of 50 000 cells/well. Ribonucleic acid (RNA) transfection occurred after 18 hours for the NC group and the experimental group (siSmad3-3). Two replicates were performed for each group. The dosages of Lipofectamine RNAiMAX and RNA used were 7.5 µL/well and 75 pmol/well, respectively. After 48 hours of transfection, the total RNA of cells was extracted for quantitative polymerase chain reaction (PCR) detection. We constructed the three siRNA gene sequences targeting Smad3 protein, and the one with the highest inhibition efficiency was screened to complete the subsequent experiment. Real-time quantitative PCR (qRT-PCR) results showed that siSmad3-3 had the highest inhibition efficiency (figure 4A). The qRT-PCR and siRNA primer sequences are shown in online supplemental file 1. Relative messenger RNA (mRNA) levels were calculated based on the Circle threshold (Ct)) values, corrected for glyceraldehyde-3-phosphate dehydrogenase (GAPDH) expression, according to the following equation: 2^–ΔCT^ (ΔCT=CT gene of interest–CT GAPDH).^16^

10.1136/wjps-2021-000289.supp1 Supplementary data

### Western blotting analysis

IEC-6 cells were inoculated into a six-well cell culture plate at a density of 50 000 cells/well. RNA transfection occurred after 18 hours for the NC group and the experimental group (siSmad3-3). Two replicates were performed for each group. The dosages of Lipofectamine RNAiMAX and RNA used were 7.5 µL/well and 75 pmol/well, respectively. After 48 hours of transfection, proteins were obtained from those IEC-6 cells. The steps for western blotting are referenced in the paper by Almoiliqy *et al*.^17^

### VEGF test

IEC-6 cells were inoculated into two 24-well cell culture plates. RNA transfection occurred after 18 hours for the NC group and the experimental group (siSmad3-3). The experiment was done in duplicates per group. The dosages of Lipofectamine RNAiMAX and RNA used were 1.5 µL/well and 15 pmol/well, respectively. After 48 hours of transfection, intracellular VEGF and extracellular VEGF were determined using enzyme linked immunosorbent assay (ELISA) kits according to the manufacturer’s instructions.

### Cell proliferation assay

IEC-6 cells were inoculated into 96-well plates at a density of 3000 cells/well. RNA transfection occurred after 18 hours for the NC group and the experimental group (siSmad3-3). Each experiment was repeated three times per group. The dosages of Lipofectamine RNAiMAX and RNA used were 0.3 µL/well and 3 pmol/well, respectively. After 72 hours of transfection, the medium of each well was replaced with DMEM complete medium containing 10% CCK8 and the plates were further incubated for 1 hour. The absorption values at 450 nm were detected using an enzyme labeling instrument. The formula for cell viability (%)=[OD450 (Smad3)−OD450 (blank)]/[OD450 (nc)−OD450 (blank)]×100%.

### Cell migration assay

IEC-6 cells were inoculated into 24-well cell culture plates (1×10^5^ cells/well). Cell transfection was the same as cell proliferation assay. The dosages of Lipofectamine RNAiMAX and RNA used were 1.5 µL/well and 15 pmol/well, respectively. After 48 hours of transfection, 20 000 cells were collected and cultured on the upper chamber of a Transwell in 100 µL of medium with no serum. Three Transwell plates were used for each group. Medium containing 10% of serum (600 µL) was added to the lower compartment. After 24 hours, the chamber was fixed with 4% paraformaldehyde, and then the cells were gently removed from the upper membrane. The cells in the lower membrane were stained with DAPI (2-(4-amidinophenyl)-6-indolecarbamidine dihydrochloride). The cells of the stained lower membrane were photographed using a fluorescence microscope. Five fields of view were randomly selected for each compartment using a 20× objective lens. The number of cells in each field of view was counted using cell counting software, and the average number counted in five fields per well was used for data analysis.

### EMT detection

IEC-6 cells were inoculated into 24-well cell culture plates at 3000 cells/well. Cell transfection was the same as cell proliferation assay. Two experiments were done per group. The dosages of Lipofectamine RNAiMAX and RNA used were 0.75 µL/well and 7.5 pmol/well, respectively. After 48 hours of transfection, EMT markers E-cadherin and vimentin were diluted (1:200) and 100 µL/well of the mixture were added to the appropriate group. Overnight incubation at 4°C followed. The solution was discarded and the cells were washed with PBS five times. FITC (fluorescein isothiocyanate)-labeled secondary antibody was diluted at 1:200, and 100 µL of this dilution were added to each well, followed by incubation for 1 hour. At room temperature, the secondary body solution was discarded, and the cells were washed with PBS five times. Then the cells were stained with DAPI for 3 min and images were taken using a fluorescence microscope.^18^

### Statistical analysis

GraphPad Prism V.7.0 and SPSS V.23.0 were used for data analysis. All values are presented as mean±SD (SD,‾x±s). χ^2^ test and t-test were used to compare means between the groups. Those that do not conform to normal distribution are represented by M (Q1, Q3) and Mann-Whitney U test is adopted. P values of less than 0.05 indicated statistically significant differences.

## Results

### Clinical and pathological features of postnecrotizing enterocolitis stricture

**Figure 1:**
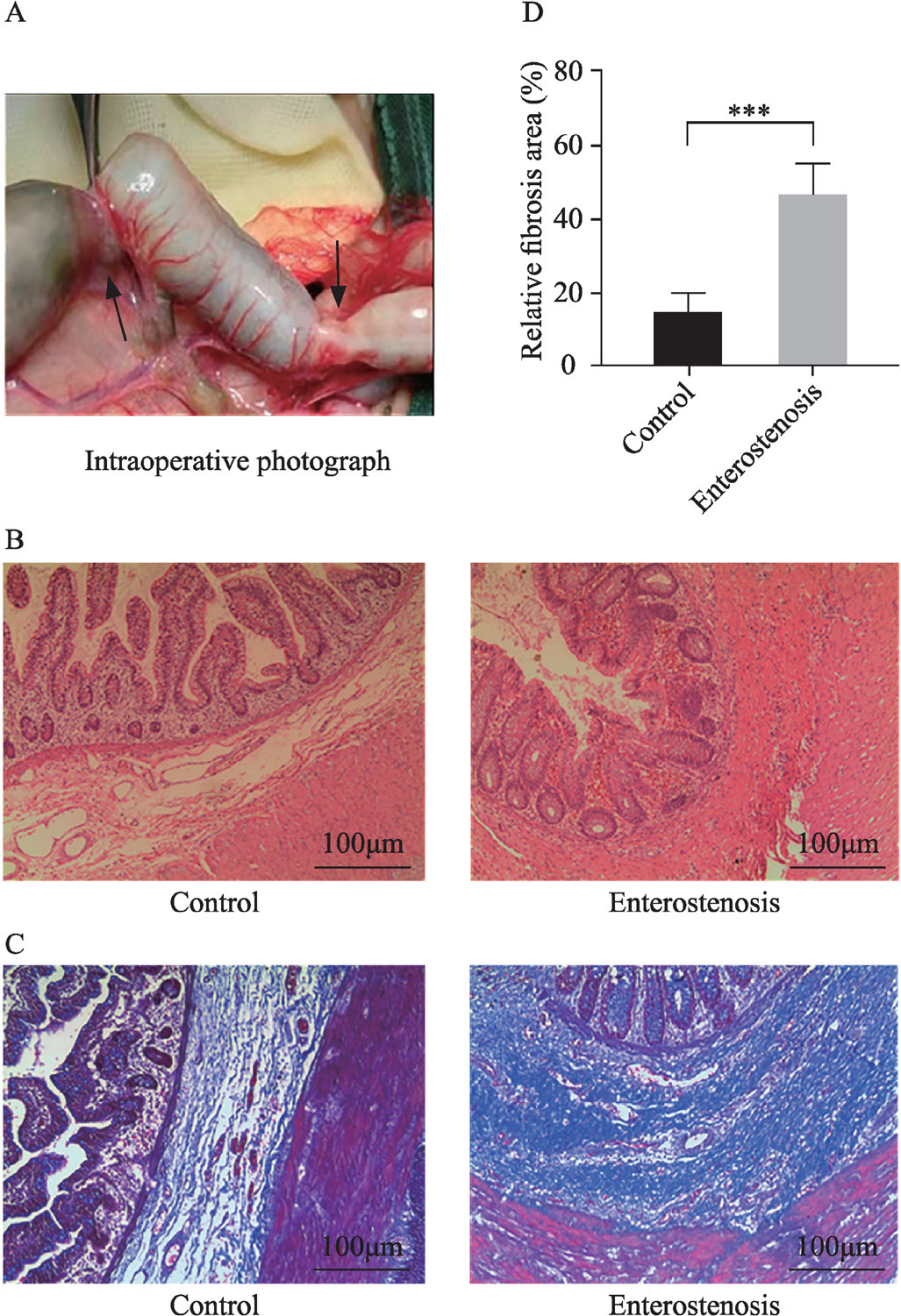
The main pathological change of postnecrotizing enterocolitis stricture was intestinal fibrosis. (A) Intraoperative photograph of postnecrotizing enterocolitis stricture. The photo shows the distal ileum and the ascending colon strictures (indicated by the black arrows). (B) H&E (hematoxylin-eosin) staining of postnecrotizing enterocolitis stricture. The enterostenosis group shows that the intestinal lumen became smaller and the submucosa was fibrous (fibers are shown in red, 5×10). (C) Masson staining of postnecrotizing enterocolitis stricture. The enterostenosis group shows that the collagen fiber deposition was obvious in the submucosa and muscular layer (collagen fibers are shown in blue, 5×10). (D) Comparison of the submucosal fibrotic area between the two groups. The enterostenosis group was significantly higher than the control group. ***P<0.0001, compared with the control group (n=56).

Of the 56 children, 35 were male, 21 were female, 50 were premature infants, and 6 were term infants. The average birth weight of the 56 children was 1750.36±65.81 g, and the average weight at surgery was 2835.38±60.76 g. The operative time of children with postnecrotizing enterocolitis stricture ranged from 18 days to 115 days after the occurrence of clinically confirmed NEC, with an average of 39.15±21.51 days. The main locations of intestinal stenosis were the terminal ileum in 11 cases, the ileocecal junction in 12 cases, and the colon in 33 cases (figure 1A).

### Pathological characteristics of NEC secondary to intestinal stenosis

H&E images showed that the lumen of the stenosis tube was narrow and obvious, and the mucosal layer was atrophic, showing collagen fiber deposition and inflammatory cell infiltration in the submucosa (figure 1B). Masson staining showed that the collagen fibers in the submucosa and myometrium of the narrow intestinal duct were significantly proliferated compared with the control group (figure 1C, D). The results of H&E and Masson staining confirmed that intestinal fibrosis played a role in intestinal stenosis.

### Expression characteristics of Smad3 protein in narrow intestinal tissue

**Figure 2:**
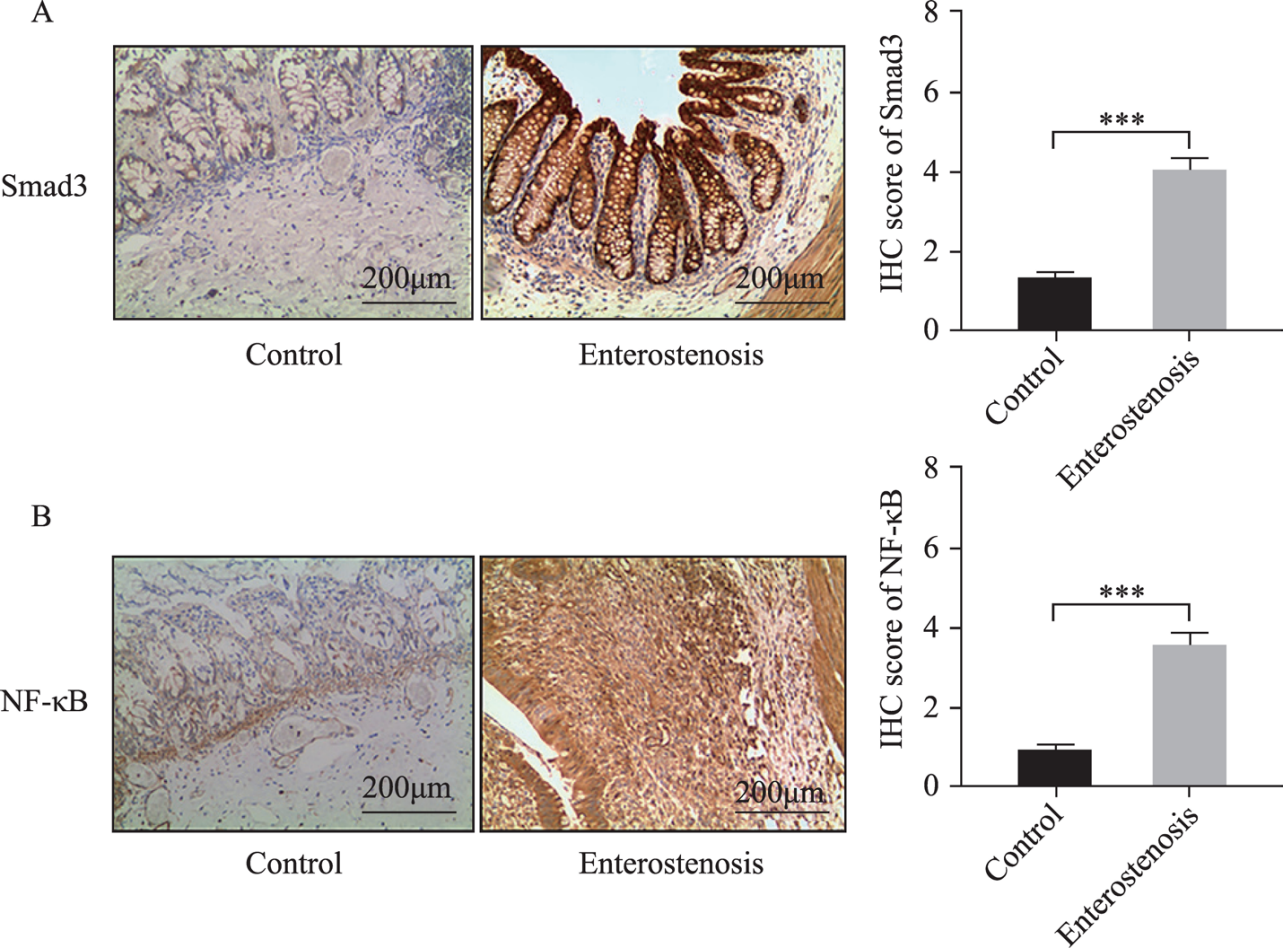
Expression of Smad3 and NF-κB proteins in the two groups. (A) Positive expression of Smad3 protein in the two groups. The brownish yellow granules are positive expression of Smad3 protein. By immunohistochemical semiquantitative score, the mean score of the enterostenosis group was significantly higher than the control group. ***P<0.0001, compared with the control group, 10×10 (N=56). (B) Positive expression of NF-κB protein in the two groups. The brownish yellow granules are positive expression of NF-κB protein. By immunohistochemical semiquantitative score, the mean score of the enterostenosis group was significantly higher than the control group. ***P<0.0001, compared with the control group,10×10 (n=56). IHC, immunohistochemistry; NF-κB, nuclear factor kappa B; Smad3, mothers against decapentaplegic homolog 3.

The expression of Smad3 protein in NEC secondary to intestinal stenosis was higher than that in the control group (66.07% (37 of 56) vs 23.21% (13 of 56), p<0.05) (figure 2A). The expression of NF-κB protein in NEC secondary to intestinal stenosis was higher than that in the control group (60.71% (34 of 56) vs 10.07% (9 of 56), p<0.05) (figure 2B). The expression of Smad3 protein and NF-κB protein in the enterostenosis group was not significantly associated with gender, preterm birth, and site of stenosis (table 1). A positive expression of Smad3 protein was positively correlated with a positive expression of NF-κB protein (r=0.659, p<0.001) (table 1).

**Table 1:** Association between clinical characteristics and Smad3 and NF-κB protein expression

General clinical features		Smad3		P value	NF-κB		P value
		+	−		+	−	
Gender	M	23	12	0.942	19	16	0.203
	F	14	7		15	6	
Weeks of gestation	<37	33	17	0.974	31	19	0.570
	≥37	4	2		3	3	
Stenosis	Ileum	15	7	0.855	13	9	0.942
	Ileocecal	4	3		4	3	
	Colon	18	9		17	10	
NF-κB	+	31	3	0.000			
	−	6	16				

F, female; M, male; NF-κB, nuclear factor kappa B; Smad3, mothers against decapentaplegic homolog 3.

**Figure 3:**
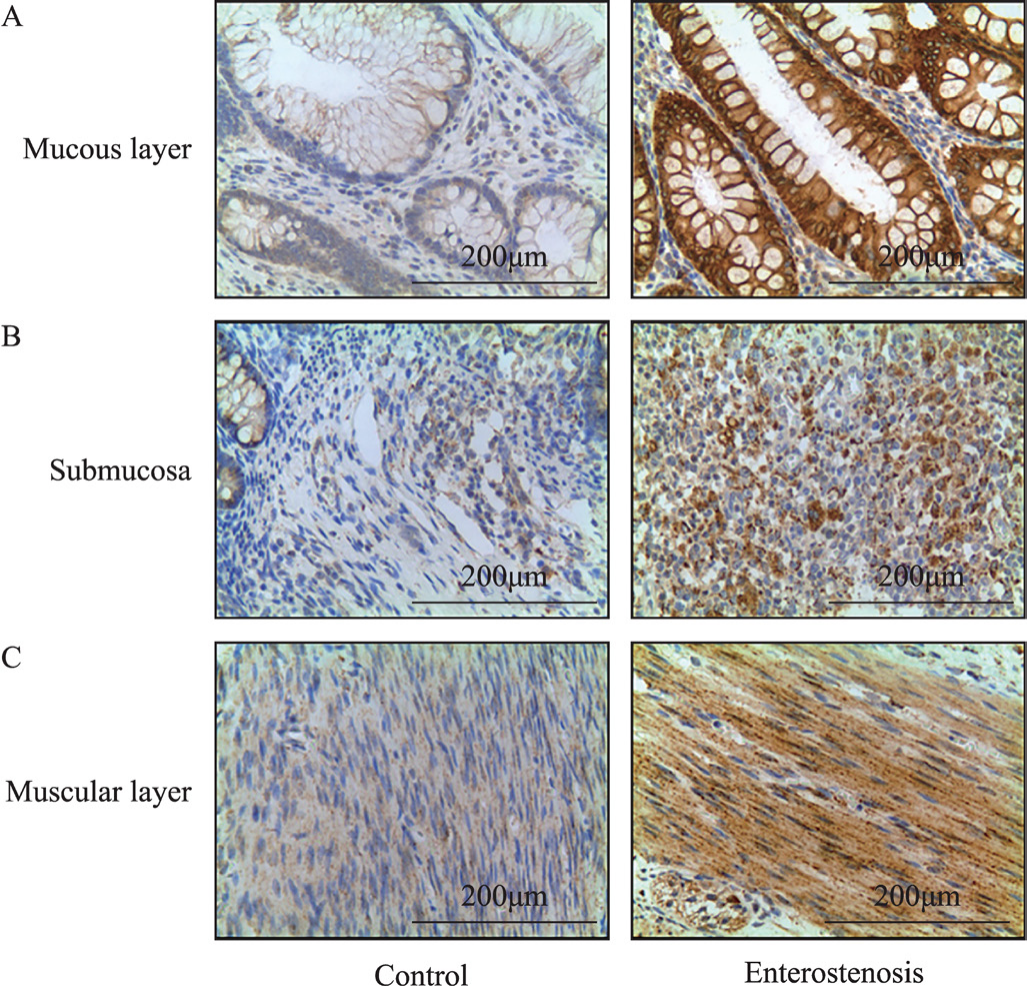
Expression of Smad3 protein in the mucosa, submucosa and muscularis in the two groups. (A) Positive expression of Smad3 protein in the mucous layer. The enterostenosis group was significantly higher than the control group. (B) Positive expression of Smad3 protein in the submucosa. The enterostenosis group was significantly higher than the control group. (C) Positive expression of Smad3 protein in the muscular layer. The enterostenosis group was significantly higher than the control group. Smad3, mothers against decapentaplegic homolog 3.

Analysis of the AOD of Smad3 protein expression in the two groups showed that the Smad3 protein expression in the narrow intestinal tissues was significantly higher than the control group (table 2). Smad3 protein was expressed in the mucosa, submucosa, and muscularis of the narrow intestine, and the expression of Smad3 protein was highest in the mucosa (figure 3).

**Table 2:** AOD of Smad3 protein positive expression in the mucosa, submucosa, and muscularis between the two groups

	Control group (n=13)	Observed group (n=37)	P value
Mucosa layer	0.263 (0.243, 0.298)	0.550 (0.516, 0.612)	0.000
Submucosa	0.238 (0.211, 0.267)	0.387 (0.366, 0.404)	0.000
Muscular layer	0.198 (0.187, 0.211)	0.319 (0.289, 0.329)	0.000

AOD, average optical density; Smad3, mothers against decapentaplegic homolog 3.

The area of fibrosis (50.42%±7.55%) in children with a positive Smad3 protein expression in the narrow intestinal duct increased by 38.53%±3.39% (p=0.000). Children with overexpression of Smad3 protein have more severe intestinal fibrosis, and Smad3 protein was involved in the process of intestinal fibrosis.

### Effect of Smad3 on NF-κB/TGF-β1/TNF-α/ZO-1/VEGF mRNA and protein expression in IEC-6 cells

**Figure 4:**
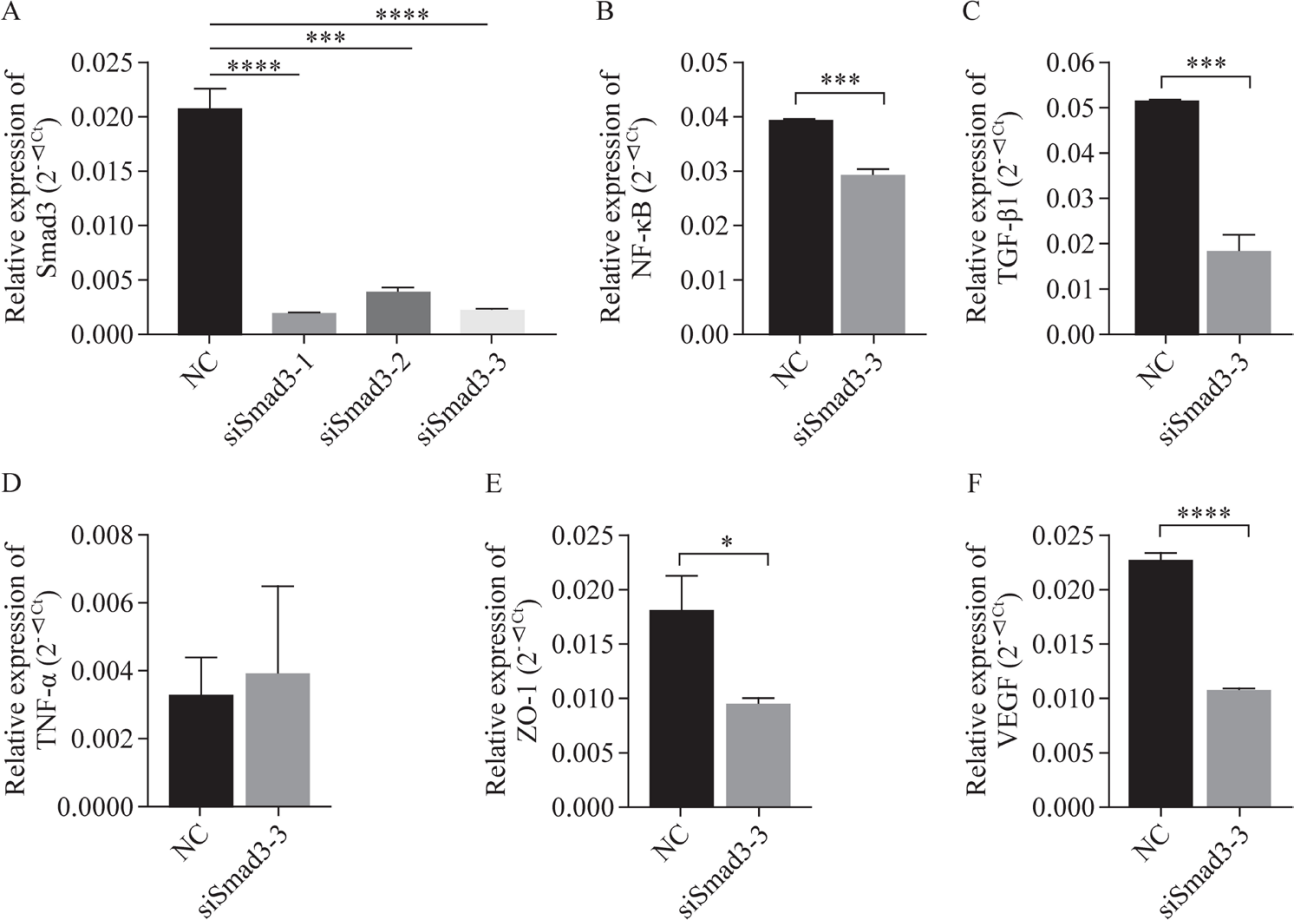
Effect of Smad3 protein on the expression of fibrosis-related indices mRNA in IEC-6 cells. (A) Smad3 mRNA expression in IEC-6 cells was inhibited by three siRNAs. The sequence siSmad3-3 was selected for subsequent experiment. (B) Expression of NF-κB mRNA in IEC-6 cells. (C) Expression of TGF-β1 mRNA in IEC-6 cells. (D) Expression of TNF-α mRNA in IEC-6 cells. (E) Expression of ZO-1 mRNA in IEC-6 cells. (F) Expression of VEGF mRNA in IEC-6 cells. *P<0.05,**P<0.01, ***P<0.001, ****P<0.0001, compared with the NC group (n=3). IEC-6, crypt epithelial cells of rat small intestine; mRNA, messenger RNA; NC, negative control; NF-κB, nuclear factor kappa B; siRNA, small interfering RNA; Smad3, mothers against decapentaplegic homolog 3; TGF-β1, transforming growth factor-β1; TNF-α, tumor necrosis factor-α; VEGF, vascular endothelial growth factor; ZO-1, zonula occludens-1.

After inhibiting the expression of Smad3 in IEC-6 cells, the mRNA levels of NF-κB (figure 4B), TGF-β1 (figure 4C), ZO-1 (figure 4E), and VEGF (figure 4F) decreased, and the mRNA level of TNF-α has no significant differences (figure 4D).

**Figure 5:**
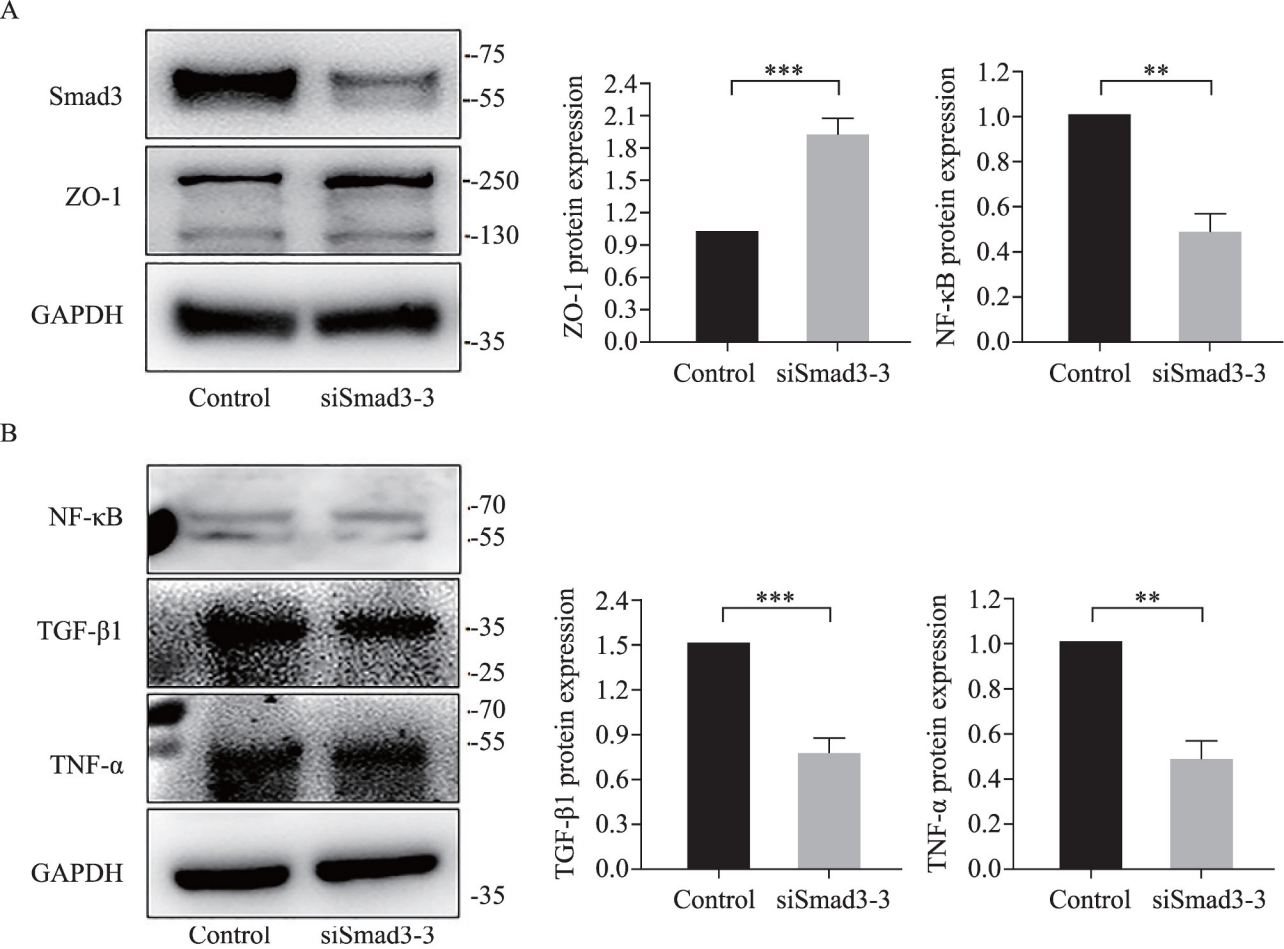
Effect of Smad3 on the expression of fibrosis-related proteins in IEC-6 cells. (A) After inhibiting Smad3 mRNA expression in IEC-6 cells, the Smad3 protein expression decreased and the ZO-1 protein expression increased. (B) After inhibiting Smad3 mRNA expression in IEC-6 cells, the expression of NF-κB, TGF-β1 and TNF-α proteins decreased. **P<0.01, ***P<0.001, compared with the NC group (n=3). GAPDH, glyceraldehyde-3-phosphate dehydrogenase; IEC-6, crypt epithelial cells of rat small intestine; mRNA, messenger RNA; NC, negative control; NF-κB, nuclear factor kappa B; Smad3, mothers against decapentaplegic homolog 3; TGF-β1, transforming growth factor-β1; TNF-α, tumor necrosis factor-α; ZO-1, zonula occludens-1.

After inhibiting the expression of Smad3 protein in IEC-6 cells (figure 5A), the expression of NF-κB, TGF-β1, and TNF-α proteins decreased (figure 5B) and the expression of ZO-1 protein increased (figure 5A). There was no significant difference in the expression of extracellular VEGF protein, and the expression value was lower than the lower limit of the ELISA kit. The expression of intracellular VEGF protein in the siSmad3 group (9.012±0.2085 pg/mL) increased compared with the NC group (7.04±0.2277 pg/mL; p=0.0007 compared with the NC group, n=4). Smad3 can regulate the expression of related proteins on inflammatory signals in IEC-6 cells.

### Effect of Smad3 protein on IEC-6 cell EMT

**Figure 6:**
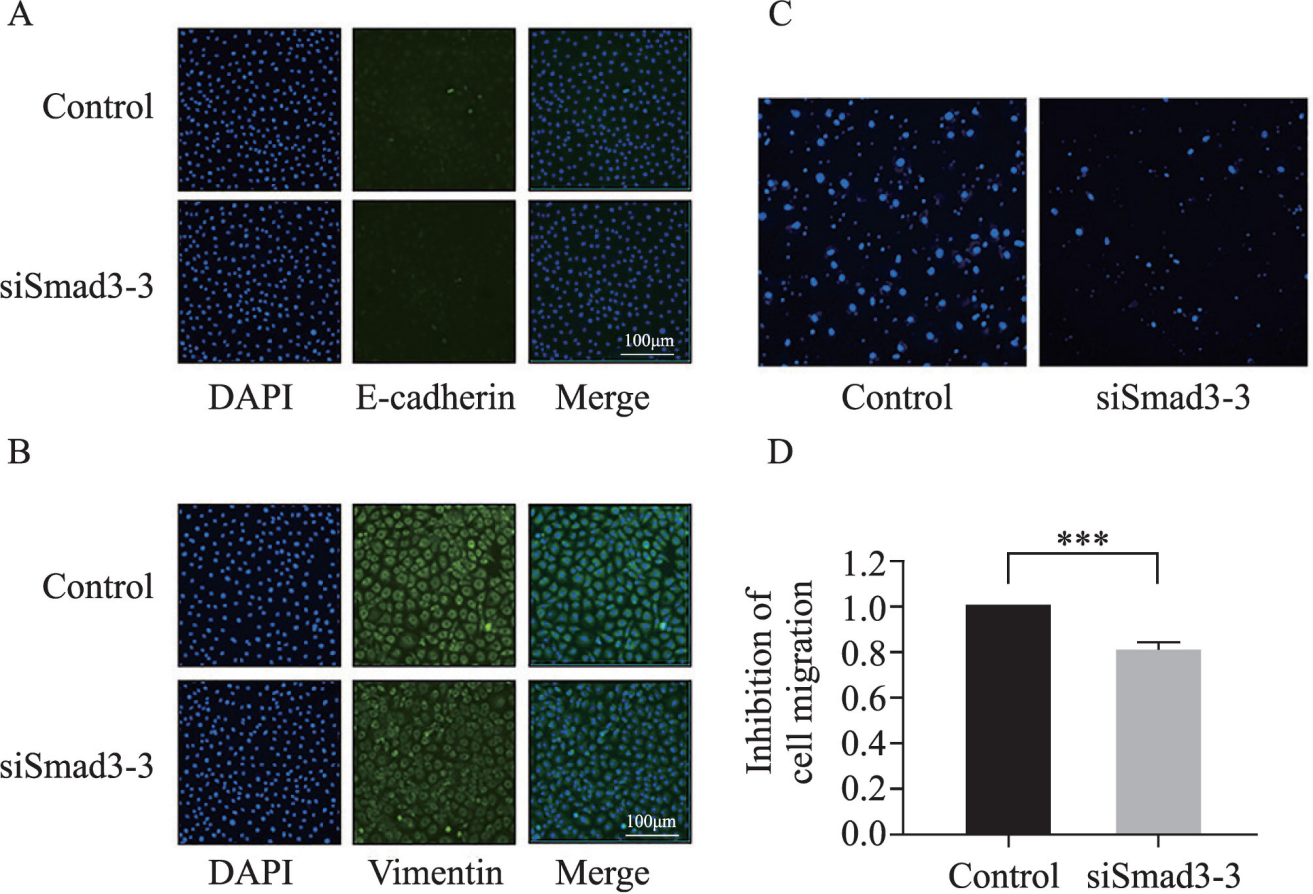
Effect of Smad3 on EMT and migration of IEC-6 cells. (A) The effect of Smad3 on the expression of E-cadherin in IEC-6 cells was observed under fluorescence microscope (200×). (B) The effect of Smad3 on vimentin expression in IEC-6 cells was observed under fluorescence microscopy (200×). (C) The effect of Smad3 on the migration of IEC-6 cells was observed under fluorescence microscope (200×). (D) Statistical diagram of inhibition efficiency of Smad3 on IEC-6 cell migration. After inhibiting Smad3 mRNA expression in IEC-6 cells, the ability of cells to migrate is reduced. ***P<0.001, compared with the NC group (n=5). DAPI, (2-(4-amidinophenyl)-6-indolecarbamidine dihydrochloride; EMT, epithelial–mesenchymal transition; IEC-6, crypt epithelial cells of rat small intestine; mRNA, messenger RNA; NC, negative control; Smad3, mothers against decapentaplegic homolog 3.

EMT in intestinal epithelial cells is an important mechanism of intestinal fibrosis and an important source of intestinal fibroblasts. After inhibiting the expression of Smad3 in IEC-6 cells, there was no significant change in the expression of EMT markers E-cadherin and vimentin (figure 6A, B). Smad3 had no significant effect on EMT of IEC-6 cells.

### Effect of Smad3 on gastrointestinal mucosal barrier function

The integrity of gastrointestinal mucosal barrier function can prevent the occurrence of intestinal fibrosis. Proliferation and migration of intestinal epithelial cells play a positive role in maintaining the complete gastrointestinal mucosal barrier function. After inhibiting the expression of Smad3 in IEC-6 cells, the migration ability of IEC-6 cells decreased and the difference was statistically significant (figure 6C, D). The cell viability of siSmad3 group was 78.82%±1.046%, lower than the NC group (p=0.0003, n=4). Smad3 can promote the proliferation and migration of intestinal epithelial cells and participate in the process of intestinal fibrosis.

## Discussion

Intestinal stenosis is a common secondary NEC disease, and its main clinical manifestations are abdominal distension, feeding intolerance, and repeated infection after treatment in children with NEC.^3^ The clinical incidence of NEC secondary to intestinal stenosis is hidden, and surgical treatment is needed to remove the intestinal canal in the stenosis segment.^2^ The mechanism of NEC secondary to intestinal stenosis remains unclear. The most likely mechanisms are the following: the inflammatory response of the intestinal wall leads to the deposition of ECM and to an abnormal expression of collagen fibers, which lead to lumen stenosis.^19^ Another possible factor is the mechanical compression of the adhesion cord, mesenteric thrombosis, intestinal wall ischemia, and other factors, leading to ischemic injury in the intestinal tissue.^20^ The results of H&E and Masson staining showed that the narrow segment of the intestinal tube was characterized by an obvious fine lumen, atrophy of the mucosal layer, an excessive expression of collagen fiber in the submucosa, infiltration of inflammatory cells, a thickening of the myometrium, and an excessive expression of collagen fibers in the myometrium. This result is similar to the pathological features of IBD-induced intestinal fibrosis.^21^ Thus, it can be inferred that intestinal fibrosis played an important role in NEC secondary to intestinal stenosis.

Common IBD is the end result of repeated chronic inflammation stimulation, via the following mechanisms: the inflammatory response leads to the activation of some cytokines such as TGF-β1/Smad3, inflammatory cells build-up, leading to ECM deposition, EMT, and an increase in the number of muscle fibroblasts.^22^ During the development of NEC, inflammatory factors, Toll-like receptor 4 (TLR4)-mediated NF-κB activation, TNF-α, interleukin 1 (IL-1), and other inflammatory mediators play an important role.^23 24^ This suggests that inflammatory response and some cytokines may play a similar role in NEC secondary to intestinal stenosis and IBD intestinal fibrosis. The immunohistochemical results showed that the protein expression levels of Smad3 and NF-κB were higher than those of normal intestinal tissues, and Smad3 was mainly expressed in the mucosal epithelium, submucosa, and muscular layer, suggesting that Smad3 and NF-κB proteins are likely involved in the occurrence of NEC secondary to intestinal stenosis. IEC is the largest number of intestinal epithelial cells and is an important cell for maintaining intestinal integrity and mucosal barrier function. IEC-6 cells have been used in NEC-related studies and are relatively mature for modeling. Intestinal EMT is an important mechanism in intestinal fibrosis. Combined with the high expression of Smad3 protein in mucosal epithelium, we select the IEC-6 cells as experimental cells.

TGF-β1/Smad3 is a key signaling pathway in pulmonary, hepatic, and renal fibrosis. TGF-β1 is a multifunctional cytokine participating in inflammatory infiltration, cell growth, apoptosis, differentiation, stimulating ECM formation, enhancing fibroblast viability, facilitating EMT, inhibiting collagen degradation, and more.^25^ Smad3 protein is a low downstream molecule of TGF-β1 and plays a key role in cells. It has been found in various diseases of renal fibrosis, where after the knockout of Smad3 protein expression the progression of renal fibrosis is significantly inhibited.^26 27^ Patients with NEC show an overexpression of Smad3 proteins in secondary intestinal stenosis. The area of fibrosis in the narrow bowel tissues with a positive expression of Smad3 is also increased compared with that in tissues having a negative Smad3 expression. This phenomenon is consistent with the results in renal and liver fibrosis, and it is suggested that Smad3 protein may be involved in the progression of intestinal fibrosis.

A study on renal and pulmonary fibrosis found that NF-κB is the upstream regulatory gene of TGF-β1, whose initiation can induce an increase in TGF-β1 protein expression and then promote the expression of Smad3 protein to promote fibrosis.^27 28^ There is also a negative feedback regulation mechanism. TNF-α can coordinate the TGF-β1 stimulation of EMT in IBD intestinal fibrosis, in which NF-κB signaling pathway is also involved.^29^ With the progression of NEC, an increase in TNF-α expression is detected in both the ileal tissue and systemic blood. TNF-α is one of the indicators of NEC early inflammatory response and may also be one of the factors promoting NEC secondary to intestinal stenosis.^30^ As NEC develops, NF-κB can promote the expression of many inflammatory factors, such as TNF-α, IL-1β, and IL-6, to promote NEC progression. Probiotics can reduce the activity of NF-κB by activating deacetylase SIRT1 (silent information regulator 1) to reduce the progression of NEC.^31^ Therefore, NF-κB, a key signaling pathway in the inflammatory response, is likely to promote NEC intestinal fibrosis. This study found that both NF-κB and Smad3 proteins are overexpressed in NEC secondary to intestinal stenosis, and a positive correlation between them was found. This confirms that Smad3 and NF-κB proteins are likely to promote intestinal fibrosis in NEC secondary to intestinal stenosis, and the underlying mechanism could be used for NEC prevention and mitigation. NF-κB/Smad3 signaling pathway is also involved in the repair of intestinal inflammation, eventually leading to fibrosis. When the expression of Smad3 in IEC-6 cells was inhibited in vitro, TGF-β1, NF-κB, and TNF-α protein expression in cells decreased, confirming that Smad3 may be involved in inflammatory response and intestinal fibrosis by negatively regulating the expression of TGF-β1, NF-κB, and TNF-α.

VEGF has been shown to inhibit renal fibrosis and EMT, which may be related to VEGF-blocking, TGF-β-induced Smad3 phosphorylation and upregulating Smad7 expression.^32^ In pulmonary fibrosis studies, the function of VEGF is controversial; some authors believe that VEGF can promote revascularization and accelerate pulmonary fibrosis,^33^ while others believe that VEGF can maintain the normal function of tissue structure to resist pulmonary fibrosis. VEGF can play a role in many diseases, not only in the inflammatory response, but also in tissue damage repair. This is also one of the mechanisms via which VEGF participates in tissue fibrosis.^34^ Lipopolysaccharide in vitro stimulation activates TNF-α pathways in mice to inhibit intestinal VEGF-A and VEGF receptor 2 expression, leading to a reduced intestinal microvascular production, ultimately aggravating NEC development.^35^ After Smad3 expression was inhibited, VEGF mRNA expression in IEC-6 cells decreased, while intracellular VEGF expression increased. This suggests that Smad3 protein may inhibit the expression of VEGF protein in intestinal epithelial cells to participate in NEC secondary to intestinal stenosis and that VEGF may play a protective role in NEC secondary to intestinal fibrosis. This conclusion needs further studies.

TLR4 not only activates *NF-κB* and regulates inflammatory signaling pathways to promote NEC progression, but can also affect the apoptosis, proliferation, and migration of intestinal epithelial cells to inhibit the repair of the intestinal mucosa, eventually leading to intestinal injury.^23^ Therefore, apoptosis, proliferation, and migration of intestinal epithelial cells are also important features of intestinal inflammatory fibrosis. The specific inhibition of Smad3 expression in IEC-6 cells results in a decline in IEC-6 cell proliferation and in reduced mobility, suggesting that during NEC treatment Smad3 can have a role in intestinal mucosal barrier by affecting the function of intestinal epithelial cells. The function and state of intestinal mucosal epithelial cells are important components of the mucosal barrier between intestinal epithelial cells, which is mainly maintained by tight junction proteins (ZO-1). When the state, function, and protein expression of intestinal mucosal epithelial cells are abnormal, the mucosal barrier is incomplete. Significant increase of TLR4 in the intestinal tract of premature infants can make the intestinal mucosa more susceptible to bacterial infection, damage the intestinal mucosal barrier function, and lead to occurrence and development of induced NEC.^36^ TLR4 can regulate NF-κB inflammatory signaling pathways during NEC progression. This study also found that Smad3 has similar functions as TLR4, which suggests that Smad3 proteins can promote NEC progression by affecting intestinal mucosal barrier function and the inflammatory response. On inhibition of Smad3 expression in IEC-6 cells in vitro, ZO-1 protein expression is elevated. ZO-1 proteins are important proteins that maintain tight epithelial cell junctions and have a decreased expression in NEC. This further suggests that Smad3 may inhibit the expression of ZO-1 protein and participate in the impairment of intestinal barrier function and accelerate intestinal fibrosis.

EMT is an important process of intestinal fibrosis, and intestinal fibroblasts transformed from epithelial cells are the main effector cells in fibrosis. In renal and pulmonary fibrosis, TGF-β1/Smad3 overexpression promotes EMT development and fibrosis.^8 25^ However, in IEC-6 cells, after inhibiting Smad3 expression, the expression of EMT markers E-cadherin and vimentin did not change significantly. This may be because Smad3 proteins in IEC-6 cells cannot regulate E-cadherin and vimentin expression. In a renal fibrosis study, after EMT increased, E-cadherin and ZO-1 expressions in epithelial cells decreased, and vimentin expression in interstitial cells increased, as well as their mobility, which may be related to the regulation of TGF-β1/Smad3 pathway.^37^ Cell proliferation and migration are important processes in EMT, and by inhibiting cell proliferation and migration the mechanism is related to blocking the activation of the TGF-β1/Smad2 signaling pathway.^38^ After inhibiting Smad3 protein expression, IEC-6 cell proliferation, migration, and ZO-1 protein expressions are also affected, which suggests that Smad3 proteins may also be involved in the EMT of IEC-6 cells. EMT-specific mechanism during the process of NEC development needs to be further studied.

In summary, the overexpression of Smad3 protein in NEC secondary to intestinal stenosis may promote intestinal fibrosis and participate in the development of secondary intestinal stenosis by promoting TGF-β1, *NF-κB*, and TNF-α protein expressions and inhibiting ZO-1 and VEGF protein expressions in the epithelial cells. Another possible mechanism may be related to the ability of Smad3 to promote the proliferation and migration of intestinal epithelial cells. More experimental results are needed to further explain the specific mechanism of Smad3 in intestinal fibrosis.

## Data Availability

Data are available upon reasonable request.accuracy and confirm

## References

[1] Niño DF , Sodhi CP , Hackam DJ . Necrotizing enterocolitis: new insights into pathogenesis and mechanisms. Nat Rev Gastroenterol Hepatol 2016;13:590–600. 10.1038/nrgastro.2016.119 27534694 PMC5124124

[2] Hu C , Shoujiang H , Qi Q . Clinical characteristics and surgical treatment of intestinal stenosis after necrotizing enterocolitis in neonates. Chin J Pediat Surg 2019;40:916–20.

[3] Gaudin A , Farnoux C , Bonnard A , et al . Necrotizing enterocolitis (NEC) and the risk of intestinal stricture: the value of C-reactive protein. PLoS One 2013;8:e76858. 10.1371/journal.pone.0076858 24146936 PMC3795640

[4] Liu W , Wang Y , Zhu J , et al . Clinical features and management of post-necrotizing enterocolitis strictures in infants: a multicentre retrospective study. Medicine 2020;99:e20209. 10.1097/MD.0000000000020209 32384517 PMC7220416

[5] Zhang H , Chen J , Wang Y , et al . Predictive factors and clinical practice profile for strictures post-necrotising enterocolitis. Medicine 2017;96:e6273. 10.1097/MD.0000000000006273 28272242 PMC5348190

[6] Zhao L , Zou Y , Liu F . Transforming growth factor-beta1 in diabetic kidney disease. Front Cell Dev Biol 2020;8:187. 10.3389/fcell.2020.00187 32266267 PMC7105573

[7] Suryadevara V , Ramchandran R , Kamp DW , et al . Lipid mediators regulate pulmonary fibrosis: potential mechanisms and signaling pathways. Int J Mol Sci 2020;21:4257. 10.3390/ijms21124257 32549377 PMC7352853

[8] Roehlen N , Crouchet E , Baumert TF . Liver fibrosis: mechanistic concepts and therapeutic perspectives. Cells 2020;9:875. 10.3390/cells9040875 32260126 PMC7226751

[9] Tao Q , Wang B , Zheng Y , et al . Vitamin D prevents the intestinal fibrosis via induction of vitamin D receptor and inhibition of transforming growth factor-beta1/Smad3 pathway. Dig Dis Sci 2015;60:868–75. 10.1007/s10620-014-3398-6 25326845

[10] Curciarello R , Canziani KE , Docena GH , et al . Contribution of non-immune cells to activation and modulation of the intestinal inflammation. Front Immunol 2019;10:647. 10.3389/fimmu.2019.00647 31024529 PMC6467945

[11] Becker C , Fantini MC , Neurath MF . High resolution colonoscopy in live mice. Nat Protoc 2006;1:2900–4. 10.1038/nprot.2006.446 17406549

[12] Torle J , Dabir PD , Korsgaard U , et al . Levels of intestinal inflammation and fibrosis in resection specimens after preoperative anti-tumor necrosis factor alpha treatment in patients with Crohn's disease: a comparative pilot study. Surg Res Pract 2020;2020:1–6. 10.1155/2020/6085678 PMC7054778 32149183

[13] Wu H , Liu L , Tan Q , et al . Somatostatin limits intestinal ischemia-reperfusion injury in macaques via suppression of TLR4-NF-kappaB cytokine pathway. J Gastrointest Surg 2009;13:983–93. 10.1007/s11605-009-0816-8 19184243

[14] Sun Q , Fan G , Zhuo Q , et al . Pin1 promotes pancreatic cancer progression and metastasis by activation of NF-κB-IL-18 feedback loop. Cell Prolif 2020;53:e12816. 10.1111/cpr.12816 32347623 PMC7260075

[15] Sun Y , Lian M , Lin Y , et al . Role of p-MKK7 in myricetin-induced protection against intestinal ischemia/reperfusion injury. Pharmacol Res 2018;129:432–42. 10.1016/j.phrs.2017.11.011 29154988

[16] Zhenye L , Chuzhong L , Youtu W , et al . The expression of TGF-β1, Smad3, phospho-Smad3 and Smad7 is correlated with the development and invasion of nonfunctioning pituitary adenomas. J Transl Med 2014;12:71. 10.1186/1479-5876-12-71 24636138 PMC3995298

[17] Almoiliqy M , Wen J , Xu B , et al . Cinnamaldehyde protects against rat intestinal ischemia/reperfusion injuries by synergistic inhibition of NF-κB and p53. Acta Pharmacol Sin 2020;41:1208–22. 10.1038/s41401-020-0359-9 32238887 PMC7609352

[18] Zu G , Guo J , Che N , et al . Protective effects of ginsenoside Rg1 on intestinal ischemia/reperfusion injury-induced oxidative stress and apoptosis via activation of the Wnt/β-catenin pathway. Sci Rep 2016;6:38480. 10.1038/srep38480 27910952 PMC5133596

[19] Phad N , Trivedi A , Todd D , et al . Intestinal strictures post-necrotising enterocolitis: clinical profile and risk factors. J Neonatal Surg 2014;3:44. 10.47338/jns.v3.134 26023515 PMC4420333

[20] Houben CH , A W I L , Tsui SY . Under pressure: a contribution to the pathogenesis of acquired ileal atresia. Case Rep 2013;2013:bcr2013201505. 10.1136/bcr-2013-201505 PMC3830420 24225736

[21] Bettenworth D , Rieder F , Dominik B , Florian R . Pathogenesis of intestinal fibrosis in inflammatory bowel disease and perspectives for therapeutic implication. Dig Dis 2017;35:25–31. 10.1159/000449079 28147352

[22] Silva FAR , Rodrigues BL , Ayrizono MdeLS , et al . The immunological basis of inflammatory bowel disease. Gastroenterol Res Pract 2016;2016:2097274. 10.1155/2016/2097274 28070181 PMC5192315

[23] Hackam DJ , Sodhi CP . Toll-Like receptor-mediated intestinal inflammatory imbalance in the pathogenesis of necrotizing enterocolitis. Cell Mol Gastroenterol Hepatol 2018;6:229–38. 10.1016/j.jcmgh.2018.04.001 30105286 PMC6085538

[24] Yin Y , Liu F , Li Y , et al . mRNA expression of TLR4, TLR9 and NF-κB in a neonatal murine model of necrotizing enterocolitis. Mol Med Rep 2016;14:1953–6. 10.3892/mmr.2016.5455 27357505 PMC4991741

[25] Kim KK , Sheppard D , Chapman HA . TGF-β1 Signaling and Tissue Fibrosis. Cold Spring Harb Perspect Biol 2018;10:a022293. 10.1101/cshperspect.a022293 28432134 PMC5880172

[26] Ji X , Wang H , Wu Z , et al . Specific Inhibitor of Smad3 (SIS3) Attenuates Fibrosis, Apoptosis, and Inflammation in Unilateral Ureteral Obstruction Kidneys by Inhibition of Transforming Growth Factor β (TGF-β)/Smad3 Signaling. Med Sci Monit 2018;24:1633–41. 10.12659/MSM.909236 29555895 PMC5872904

[27] Zheng Z-C , Zhu W , Lei L , et al . Wogonin ameliorates renal inflammation and fibrosis by inhibiting NF-κB and TGF-β1/Smad3 signaling pathways in diabetic nephropathy. Drug Des Devel Ther 2020;14:4135–48. 10.2147/DDDT.S274256 PMC7549498 33116403

[28] Wang Q , Wang J , Wang J , et al . HMGB1 induces lung fibroblast to myofibroblast differentiation through NF‑κB‑mediated TGF‑β1 release. Mol Med Rep 2017;15:3062–8. 10.3892/mmr.2017.6364 28339089 PMC5428737

[29] Hahn S , Nam M-O , Noh JH , et al . Organoid-based epithelial to mesenchymal transition (OEMT) model: from an intestinal fibrosis perspective. Sci Rep 2017;7:2435. 10.1038/s41598-017-02190-5 28550311 PMC5446415

[30] Cho SX , Berger PJ , Nold-Petry CA , et al . The immunological landscape in necrotising enterocolitis. Expert Rev Mol Med 2016;18:e12. 10.1017/erm.2016.13 27341512 PMC5001507

[31] Zhang K , Zhang X , Lv A , et al . Saccharomyces boulardii modulates necrotizing enterocolitis in neonatal mice by regulating the sirtuin 1/NF‑κB pathway and the intestinal microbiota. Mol Med Rep 2020;22:671–80. 10.3892/mmr.2020.11138 32626966 PMC7339617

[32] Stevens M , Neal CR , Salmon AHJ , et al . VEGF-A_165_ b protects against proteinuria in a mouse model with progressive depletion of all endogenous VEGF-A splice isoforms from the kidney. J Physiol 2017;595:6281–98. 10.1113/JP274481 28574576 PMC5621502

[33] Barratt SL , Blythe T , Jarrett C , et al . Differential Expression of VEGF-A_xxx_ Isoforms Is Critical for Development of Pulmonary Fibrosis. Am J Respir Crit Care Med 2017;196:479–93. 10.1164/rccm.201603-0568OC 28661183 PMC5564672

[34] Matkar P , Ariyagunarajah R , Leong-Poi H , et al . Friends turned Foes: angiogenic growth factors beyond angiogenesis. Biomolecules 2017;7:74. 10.3390/biom7040074 28974056 PMC5745456

[35] Yan X , Managlia E , Tan X-D , et al . Prenatal inflammation impairs intestinal microvascular development through a TNF-dependent mechanism and predisposes newborn mice to necrotizing enterocolitis. Am J Physiol Gastrointest Liver Physiol 2019;317:G57–66. 10.1152/ajpgi.00332.2018 31125264 PMC6689733

[36] Hackam DJ , Sodhi CP , Good M . New insights into necrotizing enterocolitis: from laboratory observation to personalized prevention and treatment. J Pediatr Surg 2019;54:398–404. 10.1016/j.jpedsurg.2018.06.012 29980346 PMC6344311

[37] Lu Q , Chen Y-B , Yang H , et al . Inactivation of TSC1 promotes epithelial-mesenchymal transition of renal tubular epithelial cells in mouse diabetic nephropathy. Acta Pharmacol Sin 2019;40:1555–67. 10.1038/s41401-019-0244-6 31235817 PMC7468253

[38] Griggs LA , Hassan NT , Malik RS , et al . Fibronectin fibrils regulate TGF-β1-induced epithelial-mesenchymal transition. Matrix Biol 2017;60-61:157–75. 10.1016/j.matbio.2017.01.001 28109697 PMC5438896

