# Supplementary material for: Expression and possible role of Smad3 in postnecrotizing enterocolitis stricture
Source: World J Pediatr Surg. 2022 Jan 5;5(1):e000289. doi: 10.1136/wjps-2021-000289 (PMC9716802; doi:10.1136/wjps-2021-000289)
Supplement: Supplementary data [file wjps-2021-000289supp001.pdf]

Additional materials(The primers used for RT-PCR are listed)

（1）qPCR Primer sequences are shown in the table below

Rat-ZO-1-F1	TTCGCCTGAAACAAACCCAG
Rat-ZO-1-R1	CTTGTGATACGTGCGAGGTG
Rat-VEGF-F1	GGAACTAGACCTCTCACCGG
Rat-VEGF-R1	CTCTCCCTTCATGTCAGGCT
Rat-TGFβ1-F1	GACCGCAACAACGCAATCTA
Rat-TGFβ1-R1	ACTGCTTCCCGAATGTCTGA
Rat-TNF-α-F1	TCCCAGAAAAGCAAGCAACC
Rat-TNF-α-R1	TAGACAGAAGAGCGTGGTGG
Rat-NF-kB-F1	CGTGAGGCTGTTTGGTTTGA
Rat-NF-kB-R1	TCTGCCCTCCTGACTCTACT
Rat-Smad3-F1	CATGGGCAAATGAAAGGGCT
Rat-Smad3-R1	CCAGGGTGAAGATGACAGGT
Rat-GAPDH-F1	TGCTGAGTATGTCGTGGAGTCT
Rat-GAPDH-R1	CAGTCTTCTGAGTGGCAGTGAT

（2）Three siRNA sequence of rat Smad3 was constructed

	sense（5'-3'）	antisense（5'-3'）
siSmad3-1	GUGAACACCAAGUGCAUUATT	UAAUGCACUUGGUGUUCACTT
siSmad3-2	CCAGAGCAAUAUUCCAGAATT	UUCUGGAAUAUUGCUCUGGTT
siSmad3-3	GCACAGCCACCAUGAGUUATT	UAACUCAUGGUGGCUGUGCTT
